# Diagnostic Value of Plasma miR-181b, miR-196a, and miR-210 Combination in Pancreatic Cancer

**DOI:** 10.1155/2020/6073150

**Published:** 2020-08-06

**Authors:** Gongpan Liu, Cunhua Shao, Anyun Li, Xiaobin Zhang, Xingjun Guo, Jiangong Li

**Affiliations:** ^1^Department of Hepatobiliary Surgery, Dongying People's Hospital, No. 317, Nanyi Road, Dongying City, Shandong Province 257091, China; ^2^Department of General Surgery, Dongying Honggang Hospital, No. 436, Huanghe Road, Dongying City, Shandong Province 257000, China

## Abstract

**Purpose:**

This study was aimed at investigating the roles of plasma miR-181b, miR-196a, and miR-210 in the diagnosis of pancreatic cancer (PC).

**Methods:**

Plasma samples were isolated from 40 patients with PC and 40 healthy individuals, respectively. The expression of miR-181b, miR-196a, and miR-210 was detected by qRT-PCR. The level of carbohydrate antigen 199 (CA199) was measured by an electrochemiluminescence (ECL) assay. The receiver operating characteristic (ROC) curve was used to analyze the diagnostic value of miR-181b, miR-196a, miR-210, CA199, and their combinations in PC.

**Results:**

The expression of plasma miR-181b, miR-196a, and miR-210 was significantly upregulated in PC patients. The plasma level of CA199 was also significantly increased in PC patients. The expression of miR-181b, miR-196a, and miR-210 was closely associated with lymph node metastasis, clinical stage, and vascular invasion but not correlated with age, gender, and tumor size. miR-181b, miR-196a, and miR-210 have lower AUC than CA199 in the diagnosis of PC. miR-181b+miR-210 and miR-196a+miR-210 also have lower AUC than CA199. It is worth noting that miR-181b+miR-196a+miR-210 has a higher AUC than CA199 in the diagnosis of PC.

**Conclusion:**

The combination of plasma miR-181b, miR-196a, and miR-210 had a good diagnostic value for PC.

## 1. Introduction

Pancreatic cancer (PC) is one of the most common gastrointestinal malignancies and the sixth leading cause of cancer-related death in China [1, 2]. Because early PC lacks specific clinical symptoms, most patients are already in advanced stages when they are diagnosed with PC [3]. Although the therapeutic techniques have greatly improved, the survival rate of PC patients remains poor [4]. The median survival time of PC is about 5 to 8 months, and the 5-year survival rate is only about 8% [5]. Therefore, identifying specific biomarkers is very important for the diagnosis of PC, especially for PC at an early stage.

MicroRNAs (miRNAs) are a class of small endogenous noncoding RNAs with 18-25 nucleotides in length, which can modulate gene expression at posttranscriptional level [6, 7]. More and more evidences have indicated that miRNAs exert important roles in the oncogenesis and metastasis of numerous tumors [8–10]; thus, the alteration of certain miRNAs may predict tumors to a certain extent. Under normal physiological condition, the levels of miRNAs are stable in the plasma. The abnormal expression of miRNAs has been detected in the plasma of patients with PC. Duell et al. have shown that plasma miR-10b, miR-21-5p, miR-30c, and miR-106b are upregulated in PC patients, and those miRNAs may be biomarkers for PC screening [11]. miR-181b, miR-196a, and miR-210 are three important miRNAs that were involved in the tumorigenesis of PC. Zhou et al. have shown that the plasma level of miR-181b is increased in patients with PC, which is correlated with tumor stage, lymph node metastases, and distant metastasis [12]. Xu et al. have found that exosome miR-196a is elevated in the plasma of patients with PC, which acts as a potential indicator of localized PC [13]. Ho et al. have demonstrated that circulating miR-210 level is elevated in PC patients, which may serve as a biomarker for diagnosis [14]. In addition, fecal miR-181b, miR-196a, and miR-210 are highly expressed in PC patients, and miR-181b and miR-210 may be biomarkers for PC screening [15]. However, the diagnostic value of plasma miR-181b, miR-196a, and miR-210 and their combinations in PC has not been fully elucidated.

In this study, the diagnostic roles of plasma miR-181b, miR-196a, and miR-210 in PC were investigated. The expression of plasma miR-181b, miR-196a, and miR-210 was measured by qRT-PCR. The level of the traditional tumor marker carbohydrate antigen 199 (CA199) was detected by an electrochemiluminescence (ECL) assay. In addition, the receiver operating characteristic (ROC) curve was used to determine the diagnostic value of the above plasma miRNAs in PC. We hope to reveal promising biomarkers in the diagnosis of PC.

## 2. Methods

### 2.1. Clinical Samples

Forty patients with pancreatic ductal adenocarcinoma (22 males and 18 females; 58 ± 10 years old; TNM stage: 9 I, 11 II, 13 III, and 6 IV) were recruited in our hospital from May 2016 to January 2019. The inclusion criteria were as follows: (1) first-time diagnosis; (2) no prior history of radiotherapy, chemotherapy, and other adjuvant therapy; and (3) no other malignant tumors. Forty healthy volunteers (22 males and 18 females; 60 ± 11 years old) were enrolled as the control. The plasma samples were collected from PC patients and healthy volunteers (*N* = 40). This study was approved by the Ethics Committee of our hospital in accordance with the Declaration of Helsinki. Written informed consent was obtained from patients and volunteers.

### 2.2. Quantitative Real-Time PCR (qRT-PCR)

Total RNA was extracted from plasma of PC patients and healthy volunteers using TRIzol (Invitrogen, USA). cDNA was then synthesized from total RNA using the Revert Aid First Strand cDNA Synthesis Kit (Thermo, USA). Subsequently, qRT-PCR was performed using the SYBR Green qPCR Master Mix (Thermo Scientific, USA) according to the manufacturer's protocol. U6 was used as the internal control. Primers were as follows: miR-181b: (forward) 5′-GCCGTAAAGTGCTGACAGT-3′ and (reverse) 5′-GTGCAGGGTCCGAGGTAT-3′; miR-196a: (forward) 5′-GAAGATCTTTCCTTGGCGGCGACA-3′ and (reverse) 5′-CCCAAGCTTGATGGCCCGCCTA-3′; miR-210: (forward) 5′-TATACAAGGGCAAGCTCTCTGT-3′ and (reverse) 5′-AGAGAGCTTGCCCTTGTATATT-3′; and U6: (forward) 5′-CTCGCTTCGGCAGCACA-3′ and (reverse) 5′-AACGCTTCACGAATTTGCGT-3′.

### 2.3. ECL Assay

The plasma level of CA199 was measured by the ECL assay using an electrochemical luminescence apparatus (CobasE601, Roche, Sweden). The detection threshold of CA199 was 27 U/mL.

### 2.4. Statistical Analysis

All statistical analyses were performed using SPSS 22.0 statistical software (Chicago, IL, USA). Data were presented in the form of mean ± standard deviation (SD). The two-tailed *t*-test was used for comparison between two groups, and one-way ANOVA was used for comparison among multiple groups. The chi-square test was used to compare the qualitative data. The diagnostic value of miRNAs was analyzed by the ROC curve. *P* < 0.05 was considered to be statistically significant.

## 3. Results

### 3.1. The Levels of miR-181b, miR-196a, miR-210, and CA199 Are Increased in PC Patients

**Figure 1:**
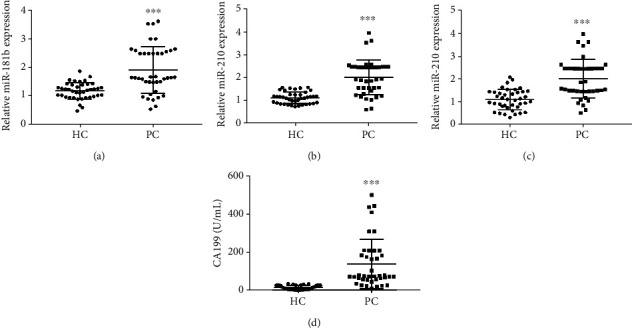
The levels of plasma miR-181b, miR-196a, miR-210, and CA199 in pancreatic cancer (PC) patients and healthy volunteers. (a) The expression of plasma miR-181b was detected by qRT-PCR. (b) The expression of plasma miR-196a was detected by qRT-PCR. (c) The expression of plasma miR-210 was detected by qRT-PCR. (d) The level of plasma CA199 was detected by the electrochemiluminescence assay. ^∗∗∗^*P* < 0.001 vs. HC group.

Forty PC patients and healthy volunteers were enrolled in this study. There were no significant differences in the age and gender between PC patients and healthy volunteers. The results of qRT-PCR showed that the expression of miR-181b, miR-196a, and miR-210 in the plasma of PC patients was higher than that in healthy volunteers (*P* < 0.001) (Figures 1(a)–1(c)). In addition, CA199 level in the plasma of PC patients was significantly higher than that in healthy volunteers (15.69 ± 11.37 vs. 139.40 ± 129.40, *P* < 0.001) (Figure 1(d)). The above results indicate that the levels of miR-181b, miR-196a, miR-210, and CA199 are increased in PC patients.

### 3.2. The Expression of miR-181b, miR-196a, and miR-210 Is Associated with Clinical Parameters in PC

The expression of miR-181b, miR-196a, and miR-210 was significantly correlated with the lymph node metastasis (*P* < 0.001, *P* < 0.01, and *P* < 0.01, respectively), clinical stage (*P* < 0.01, *P* < 0.05, and *P* < 0.01, respectively), and vascular invasion (*P* < 0.001, *P* < 0.01, and *P* < 0.01, respectively) (Table 1). However, the expression of miR-181b, miR-196a, and miR-210 had no significant correlation with the age, gender, and tumor size in PC patients (*P* > 0.05) (Table 1).

### 3.3. Plasma miR-181b, miR-196a, and miR-210 Have a Certain Diagnostic Value in PC

**Figure 2:**
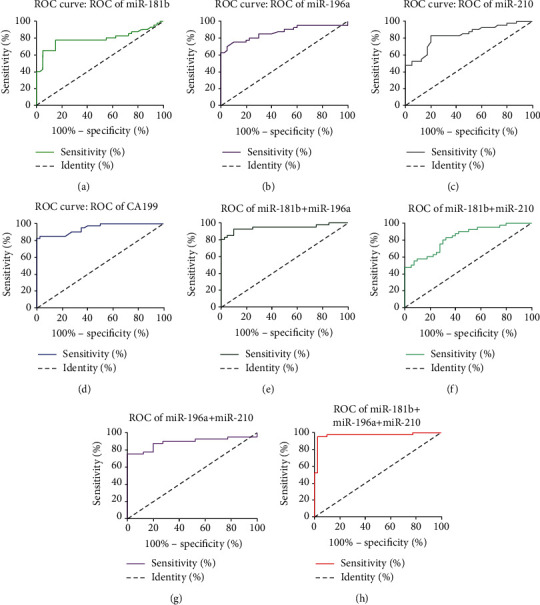
ROC curves of plasma miR-181b, miR-196a, miR-210, and CA199 in the diagnosis of PC: (a) miR-181b; (b) miR-196a; (c) miR-210; (d) CA199; (e) miR-181b+miR-196a; (f) miR-181b+miR-210; (g) miR-196a+miR-210; (h) miR-181b+miR-196a+miR-210.

The ROC curve was established to investigate the diagnostic value of plasma markers in PC (Figures 2(a)–2(h)). The results showed that miR-181b, miR-196a, and miR-210 have a lower diagnostic value (AUC) than CA199 in PC (Table 2) (all *P* < 0.05). In addition, the diagnostic value (AUC) of miR-181b+miR-210 and miR-196a+miR-210 in PC was also significantly lower than that of CA199 (Table 3) (all *P* < 0.05). It is worth noting that the combination of plasma miR-181b, miR-196a, and miR-210 (miR-181b+miR-196a+miR-210) has a higher AUC than CA199 in the diagnosis of PC (Table 3) (*P* < 0.05). To sum up, miR-181b+miR-196a+miR-210 (sensitivity: 95.00, specificity: 97.50) improved the diagnostic value of each indicator alone (Table 3).

## 4. Discussion

In recent years, the difficulty and delay in the diagnosis of PC are still a therapeutic challenge, which leads to poor prognosis. The main therapeutic methods for PC are surgery, chemotherapy, and radiotherapy. Nevertheless, the median survival time is 5 to 8 months, and the 5-year survival rate (8%) is poor [5]. In this study, we explored the diagnostic value of plasma miR-181b, miR-196a, and miR-210 in PC. We found that the combination of plasma miR-181b, miR-196a, and miR-210 has a good diagnostic value for PC.

Accumulating evidence has reported that miRNAs play important roles in PC [16–19]. Qiao et al. have shown that miR-381 is a tumor suppressor, which inhibits cell proliferation, migration, and invasion, and induces cell apoptosis in PC [20]. Zhou et al. have reported that miR-340 is downregulated in PC, which can suppress cell growth and reduce tumor size in PC [17]. Xu et al. [21] have indicated that miR-143 is lowly expressed in PC, which can promote cell apoptosis and inhibit cell migration and invasion. In addition, Xu et al. [21] have also confirmed that miR-143 expression is closely associated with the tumor size, clinical staging, and lymph node metastasis. Previous researches have determined that the expression of miR-196a and miR-210 is upregulated in the plasma of PC patients, indicating that miR-196a and miR-210 may serve as potential biomarkers for PC diagnosis [22]. Ren et al. [15] have reported that fecal miR-181b, miR-196a, and miR-210 are highly expressed in PC patients, suggesting that those miRNAs may be biomarkers for PC diagnosis. In the present study, we found that the expression of miR-181b, miR-196a, and miR-210 was closely associated with lymph node metastasis, clinical stage, and vascular invasion but not correlated with age, gender, and tumor size in patients with PC. Therefore, we speculate that miR-181b, miR-196a, and miR-210 may be used as biomarkers for PC diagnosis.

Recently, some diagnostic biomarkers in the plasma of PC patients, such as CA199, CA242, MIC-1, and lectins, are accepted for the diagnosis of PC [23–26]. Among these diagnostic biomarkers, CA199 is considered the best available biomarker for PC [23, 27, 28]. Previous studies have shown that the AUC of CA199 in the diagnosis of PC is about 0.8-0.9 [29–32]. In the present study, the level of CA199 was significantly increased in the plasma of PC patients. The AUC of CA199 in the diagnosis of PC was 0.947, which is higher than that reported previously. This phenomenon may be attributed to the differences in the enrolled subjects. The strict inclusion criteria in this study may enlarge the diagnostic value of CA199. In addition, jaundice-induced increase in CA199 may also disturb the diagnostic value evaluation. Even so, CA199 is not an ideal marker for the diagnosis of PC, especially due to the unsatisfied sensitivity. It is important to explore more effective diagnostic biomarkers for PC. In this study, the expression of miR-181b, miR-196a, and miR-210 was significantly upregulated in PC patients. ROC curves showed that miR-181b, miR-196a, and miR-210 have lower AUC than CA199 in the diagnosis of PC. Studies have confirmed that the combination of plasma miRNAs with CA199 is effective for the diagnosis of PC [23, 33]. In this study, the combination improved the diagnostic value of each indicator alone in PC. It is worth noting that miR-181b+miR-196a+miR-210 has higher AUC than CA199. These results indicate that the combination of plasma miR-181b, miR-196a, and miR-210 is a promising diagnostic strategy for PC.

## 5. Conclusions

In conclusion, the expression of miR-181b, miR-196a, and miR-210 was significantly upregulated in patients with PC. The combination of plasma miR-181b, miR-196a, and miR-210 had a good diagnostic value for PC. Our research provides a theoretical foundation for investigating novel biomarkers for the diagnosis of PC.

## Figures and Tables

**Table 1:** Correlation between the expression of plasma miR-181b, miR-196a, and miR-210 and clinical indicators in PC patients.

Clinical indicators	Case	miR-181b expression	*P* value	miR-196a expression	*P* value	miR-210 expression	*P* value
Age (y)	40						
<60	21	1.940 ± 0.833	0.7603	2.040 ± 0.808	0.9349	2.020 ± 0.851	0.9901
≥60	19	1.860 ± 0.810		2.020 ± 0.722		2.02 ± 0.861	
Gender							
Male	22	1.830 ± 0.773	0.5477	1.940 ± 0.692	0.4376	1.930 ± 0.796	0.4633
Female	18	1.990 ± 0.895		2.130 ± 0.840		2.130 ± 0.911	
Lymph node metastasis							
Yes	23	2.490 ± 0.618	0.0010^∗∗∗^	2.290 ± 0.618	0.0008^∗∗^	2.050 ± 0.511	0.0013∗∗
No	17	1.647 ± 0.635		1.428 ± 0.356		1.528 ± 0.355	
Clinical stage							
I-II	21	2.330 ± 0.768	0.0048^∗∗^	2.390 ± 0.698	0.0319^∗^	2.520 ± 0.730	0.0027^∗∗^
III-IV	19	1.620 ± 0.630		1.820 ± 0.611		1.670 ± 0.623	
Vascular invasion							
Yes	20	2.260 ± 0.594	0.0002^∗∗∗^	2.340 ± 0.479	0.0016^∗∗^	2.230 ± 0.533	0.0019∗∗
No	20	1.550 ± 0.369		1.728 ± 0.593		1.628 ± 0.348	
Tumor size (cm)							
<2	14	1.930 ± 0.884	0.9703	2.040 ± 0.838	0.9779	2.050 ± 0.973	0.9722
2-4	13	1.930 ± 0.818		2.050 ± 0.723		2.030 ± 0.801	
>4	13	1.860 ± 0.826		1.990 ± 0.764		1.980 ± 0.0.804	

Notes: ^∗∗^*P* < 0.01 and ^∗∗∗^*P* < 0.001.

**Table 2:** The diagnostic value of plasma miR-181b, miR-196a, miR-210, and CA199 in PC.

Markers	Cutoff	Sensitivity (%)	Specificity (%)	AUC (area ± Std.error)	95% CI	*P* value
miR-181b	1.45	77.50	85.00	0.789 ± 0.055^∗∗^	0.681-0.898	<0.0001
miR-196a	1.56	72.50	92.50	0.865 ± 0.044^∗^	0.779-0.951	<0.0001
miR-210	1.46	82.50	80.00	0.834 ± 0.045^∗∗^	0.745-0.923	<0.0001
CA199	41.33	82.50	99.00	0.947 ± 0.023	0.902-0.993	<0.0001

Notes: AUC: area under the curve; CI: confidence intervals. The *P* value represented the significance of ROC analysis. ^∗^*P* < 0.05, ^∗∗^*P* < 0.01 vs. CA199.

**Table 3:** The diagnostic value of plasma miRNA combinations in PC.

Tumor markers	Sensitivity (%)	Specificity (%)	AUC (area ± Std.error)	95% CI	*P* value
miR-181b+miR-196a	92.50	90.00	0.944 ± 0.029	0.887-1.001	<0.0001
miR-181b+miR-210	80.00	70.00	0.830 ± 0.045^∗∗^	0.743-0.917	<0.0001
miR-196a+miR-210	87.50	80.00	0.888 ± 0.042^∗^	0.805-0.970	<0.0001
miR-181b+196a+210	95.00	97.50	0.968 ± 0.022^∗^	0.924-1.011	<0.0001
CA199	82.50	99.00	0.947 ± 0.023	0.902-0.993	<0.0001

Notes: AUC: area under the curve; CI: confidence intervals. The *P* value represented the significance of ROC analysis. ^∗^*P* < 0.05, ^∗∗^*P* < 0.01 vs. CA199.

## Data Availability

The data used to support the findings of this study are available from the corresponding author upon request.
